# Long-term metal exposure changes gut microbiota of residents surrounding a mining and smelting area

**DOI:** 10.1038/s41598-020-61143-7

**Published:** 2020-03-10

**Authors:** Mengmeng Shao, Yi Zhu

**Affiliations:** The College of Food Science and Nutritional Engineering, China Agricultural University, Beijing, 100083 China

**Keywords:** Health care, Risk factors

## Abstract

In this epidemiologic study, 16 S rRNA sequencing was used to investigate the changes of diversity and composition profile of gut microbiota resulting from long-term exposure to multiple metals, including arsenic (As), cadmium (Cd), cuprum (Cu), lead (Pb), and zinc (Zn). Due to long-term exposure to various metals, the relative abundances of *Lachnospiraceae*, *Eubacterium eligens*, *Ruminococcaceae* UGG-014, *Erysipelotrichaceae* UCG-003, *Tyzzerella* 3, *Bacteroides*, *Slackia*, *italics*, and *Roseburia* were found to become much higher, whereas the abundance of *Prevotella 9* presented an opposite trend. Additionally, differences between males and female groups were found, such as the greater richness and evenness of bacteria for men subjected to long-term metal exposure in polluted areas. The changes of men’s microbiomes were more significant as a result of higher daily intake, mining and smelting activity, and living habits. This research presents a new theoretical basis for the correlation between long-term metal exposure and gut health for people living in contaminated areas.

## Introduction

Recently, the entire gut microecosystem has begun to be regarded as an essential organ in the human body^1^. The intestinal microbiome plays an important role, and the microorganisms contained in the microbiome are vast in number, consisting of populations of up to 100 trillion^2^. The intestinal microbiome has been proven to be profoundly responsible for maintaining human health, such as energy metabolism, environmental adaptation, immune adjustment, and even brain functions^3–5^. In addition, some related metabolic diseases (obesity, cirrhosis, hypertension, etc.) have been associated with the changed structure of the microbiome^6–8^.

The daily diet is the most critical factor in maintaining the symbiotic relationship between intestinal flora and the host throughout the host’s life^9^. An epidemiologic study has confirmed that gut microbiota can respond to various dietary styles. Rural children who consumed low-sugar, high-fiber diets were found to have higher microbial richness and biodiversity compared with European children^10^. Healey *et al*. have also found inter-individual variability in gut microbiota response to dietary interventions^11^. Some other influencing factors include age, sex, and exercise, as well as prebiotic and probiotic agents^12–14^.

16 S ribosomal RNA (rRNA) gene sequence analysis has been widely used to identify bacterial species and perform taxonomy, and massively parallel sequencing (MPS) is also commonly utilized^15^. There are nine “hypervariable regions” (V1–V9) in the sequence that can show considerable sequence diversity and are regarded as diagnostic assays for different bacteria^16^. Dethlefsen *et al*.^17^ determined the pervasive effects of antibiotics on gut microbiota by tag pyrosequencing of the 16 S rRNA V3 region. By pyrosequencing over 40,000 16 S rRNA gene V4 region amplicons per subject, links between health conditions and intestinal microbiota in elderly Irish subjects were reported^18^. Additionally, the V3–V4 regions of the 16 S rDNA gene were used in a comparison between patients with acne vulgaris and healthy controls^19^.

Dietary exposure is the major pathway of the accumulation of potential toxic metals in the body^20^. Some metals have shown different abilities to change the bacterial profile by altering its diversity and composition, and some host metabolisms have even been found to be altered^21^. Inorganic As exposure has been found to induce gut microbe perturbations, and even hepatocellular carcinoma^22^. The inhibited growth rate of the bacteria population, an altered overall gut microbial census, and an impaired gut barrier were found in mice fed with CdCl_2_ for 3 weeks^23^. Other toxic metals (Al, Pb, Co, etc.) have also been found to have negative effects on the gut microbiome^24^. In addition, the deficiency or excess of essential elements such as Zn and Fe have also exhibited the capability to shift gut microbiota composition and function^25–27^.

Hezhang County in Guizhou Province, China is most famous for its Pb-Zn mine^28^. The mineral mine in this area consists of mountain deposits, which account for over 70% of the plowed soils. Because production and marketing principally occur locally, contaminated crops, vegetables, and animal foodstuff cause mineral metal accumulations in the bodies of local residents within the region^29,30^. In a previous study by the authors, exposure levels and the harmful health effects of multiple metals in local subjects were reported^31^. However, the effect of multiple metals on the human microbiome is still unclear. Therefore, this paper reports an investigation of the synergistic effects of multiple metals on human gut microbiota, and fills in the gaps of the epidemiological studies in the crosstalk between heavy metals and intestinal flora.

## Results

### Individual information for the two study areas

From screening using a questionnaire, a total of 40 healthy residents took part in the stool collection activity (Table 1). Twenty participants (11 female and 9 male) from Maomaochang Village were assigned the group label T (Test), and the remaining participants (13 female and 7 male) from Yemachuan Village were given the group label CK (Control check). Compared to the population of CK, people in T were younger on average (Student’s Test, *p* < 0.01). The mean body mass index (BMI) value of the two groups was equal. Due to differences of intestinal flora between men and women, four groups divided by gender (labels: TM and CKM, TF and CKF) were used for comparative analysis^20^. The mean age of men in T was lower than that in CK, and there were no significant differences in body weight. Furthermore, the percentages of smokers and drinkers in both T and TF were larger than those in the corresponding groups from the control village population; nevertheless, the population of male smokers was larger in the control area.

**Table 1:** Individual information on volunteers from the two areas.

		T	CK
Age (Mean ± SD)	Male	52.89 ± 10.06	60.57 ± 6.97
	Female	49.91 ± 9.77	50.54 ± 6.13
	Total	51.25 ± 10.01	54.05 ± 8.02
BMI (Mean ± SD)	Male	21.18 ± 2.83	21.14 ± 2.74
	Female	23.70 ± 3.37	23.79 ± 3.49
	Total	22.56 ± 3.38	22.90 ± 3.47
Smoker	Male	44%	14%
	Female	9%	0
	Total	25%	5%
Drinker	Male	44%	57%
	Female	9%	0
	Total	25%	20%

SD, standard deviation; T, Test; CK, Control check.

### Long-term exposure to various heavy metals changes the diversity of gut microbiota

**Figure 1:**
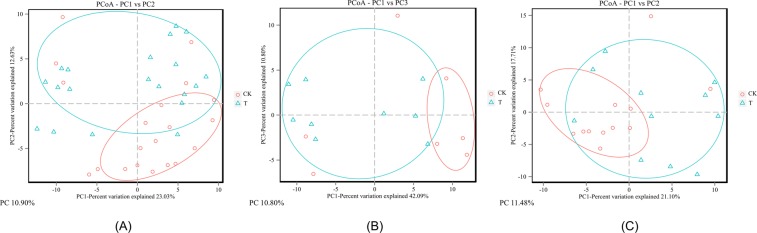
Principal coordinate analysis (PCoA) of the fecal bacterial compositions of these groups. (**A**) The discrete degrees of samples in groups CK and T. (**B**) The discrete degrees of male samples in the two groups. (**C**) The discrete degrees of female samples in the two groups.

A total of 1,449,804 (1.45 million) 16 S rRNA reads were generated from the stool samples in T, with an average of 74,690 (±5,962 SD) reads in TM and 70,690 (±5,322 SD) reads in TF. A slightly lower number of reads (1,400,222) was detected in CK, with an average of 72,203 (±5,100 SD) reads in CKM and 68,830 (±6,594 SD) reads in CKF. Rarefaction analysis was conducted on the number of OTUs per sample, as presented in Figure S1. The plots tend towards a plateau but do not reach it, suggesting that new OTUs may occur as the number of samples increases. In contrast, the species accumulation curves all reach peaks, suggesting that new species may emerge as the sequencing depth increases, while causing little effect on the diversity of intestinal flora. The analysis of α-diversity metrics revealed the effects of multiple metals on the microbiota (Table 2). There were no significant differences in community richness (Chao1 and ACE indices) between T and CK. However, significantly higher values of the Shannon-Wiener Index, and much lower values of the Simpson Index, were found in group T, suggesting that the multiple metals accumulated in the participants’ bodies, thereby increasing the bacteria richness and evenness (Wilcox Test, *p* < 0.05). Similarly, differences between the groups (TM and CKM, TF and CKF) were also compared. The microbiota of the TM subjects had observably larger Shannon-Wiener index values compared with those of CKM (Wilcox Test, *p* < 0.01). Additionally, much lower values of the Simpson Index were also found for TM (Wilcox Test, *p* < 0.05). These findings all imply that long-term metal exposure increased the richness and evenness of bacteria for men who live in the polluted area. No significant changes were found between the female populations. As another means of identifying changes in the microbiota induced by metal exposure, PCoA was performed for all the samples in this study using the unweighted UniFrac distance method (Fig. 1); a higher clustering of the microbial population in CK than in T, which had greater dispersion, was observed. The same discrete situations were also found in the gender-based groups. These results suggest that the metal exposures changed the gut microbial communities of the local residents, with varying effects on different individuals. The PCA analysis also shows the qualitatively similar results in these groups (Fig. S2).

**Table 2:** Decreased α-diversity in the Test group compared with CK.

		Chao1	ACE	Shannon	Simpson
Male	TM	413.61 ± 18.58	412.65 ± 18.54	4.26 ± 0.14	0.04 ± 0.01
	CKM	408.42 ± 17.07	402.94 ± 17.98	3.34 ± 0.75^**^	0.15 ± 0.13^*^
Female	TF	406.03 ± 24.70	400.00 ± 23.07	4.04 ± 0.38	0.06 ± 0.03
	CKF	412.30 ± 15.44	407.90 ± 14.24	3.97 ± 0.28	0.06 ± 0.02
Total	T	409.44 ± 21.94	405.69 ± 21.60	4.14 ± 0.31	0.05 ± 0.03
	CK	410.94 ± 15.69	406.17 ± 15.36	3.75 ± 0.57^*^	0.09 ± 0.09^*^

Asterisks (**) and (*) indicate significant difference (p < 0.01 and p < 0.05, respectively).

T, Test; CK, Control check; TM, Test Male; CKM, Control check Male; TF, Test Female; CKF, Control check Female.

### Metal-induced change in microbial composition

**Figure 2:**
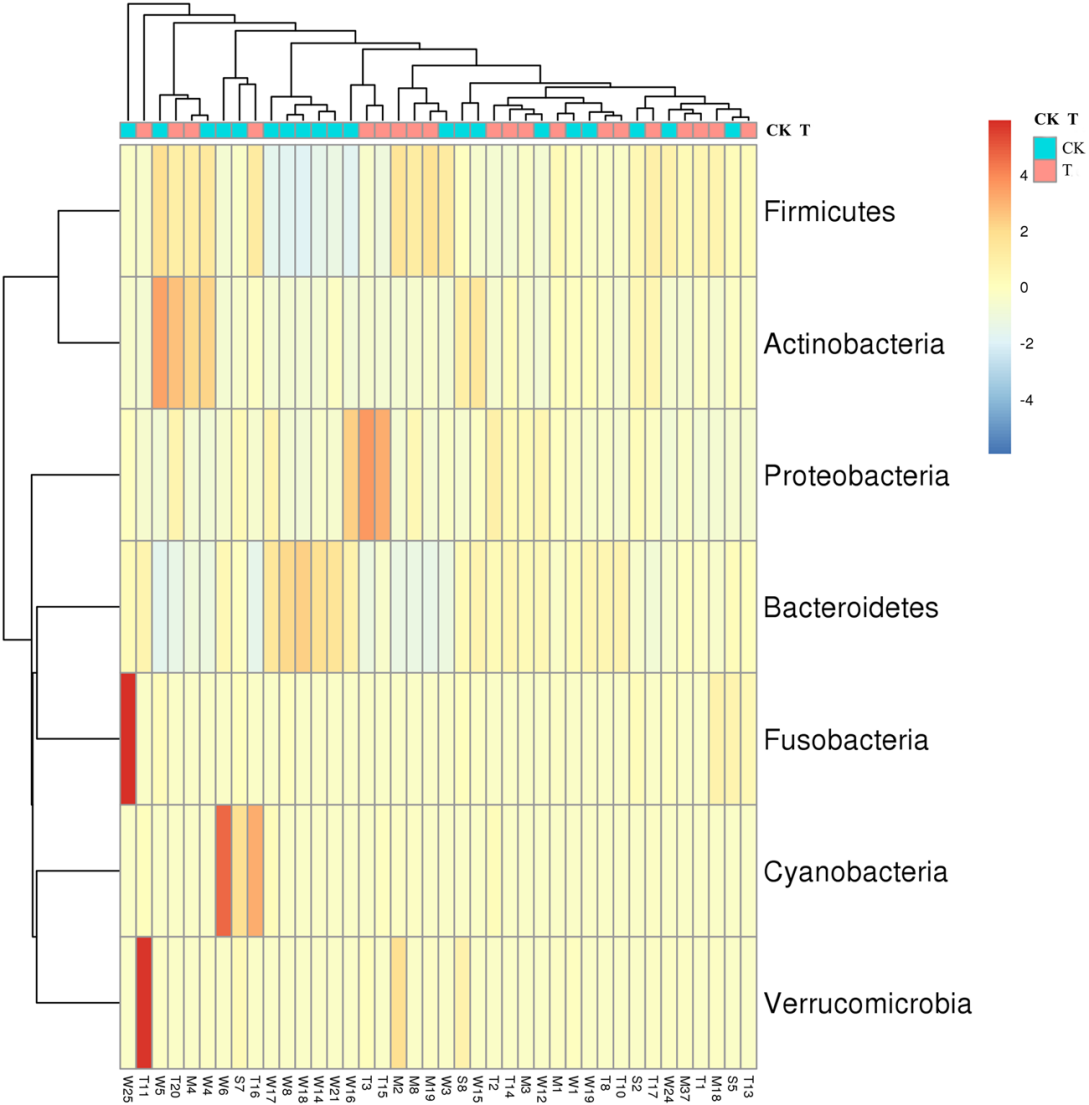
A heatmap dendrogram at the phyla level. The values of OTUs were log2 transformed. Red and blue dots after each row represent samples from groups T and CK, respectively.

**Figure 3:**
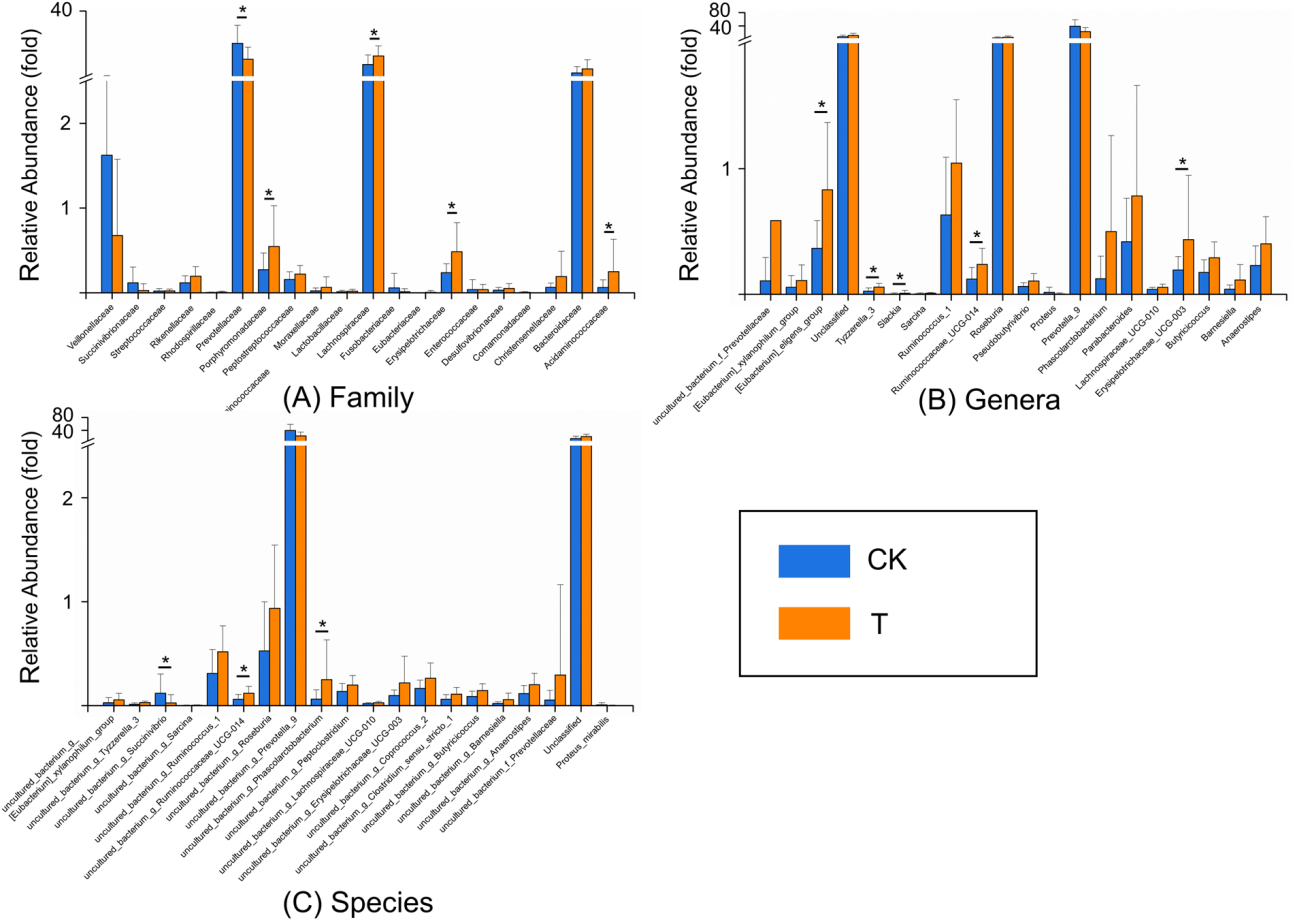
Differences in the relative abundances of the most abundant (**A**) family, (**B**) genera, and (**C**) specie in groups T and CK. Blue indicates taxa enriched in CK, and orange indicates taxa enriched in T.

**Figure 4:**
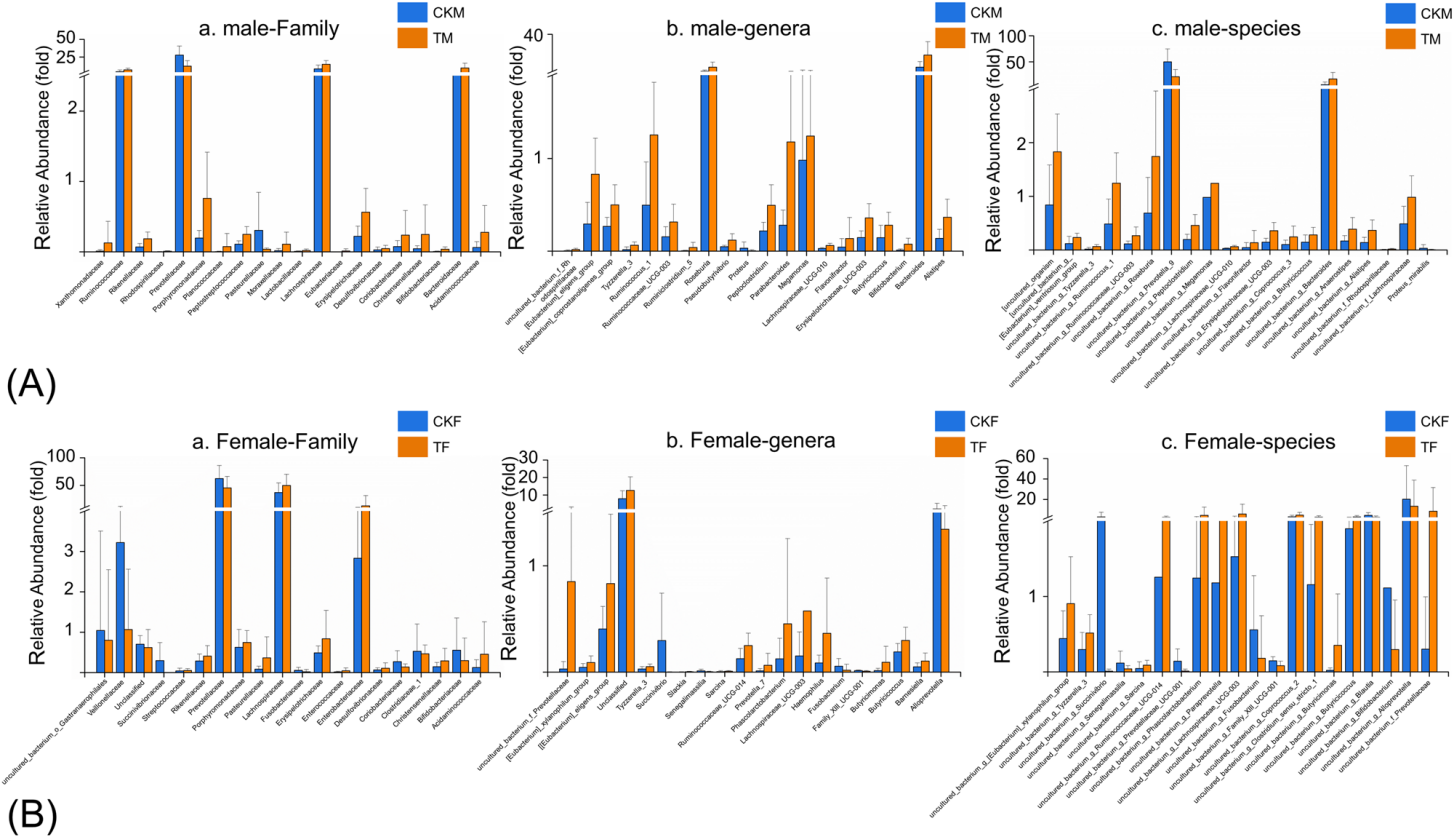
Differences in the relative abundances at different levels analyzed in the gender-based groups (male at (**A**), female at (**B**); the most abundant (a**)** family, (b) genera, and (c) specie. Blue indicates taxa enriched in CK of male or female, and orange indicates taxa enriched in T of male or female.

**Figure 5:**
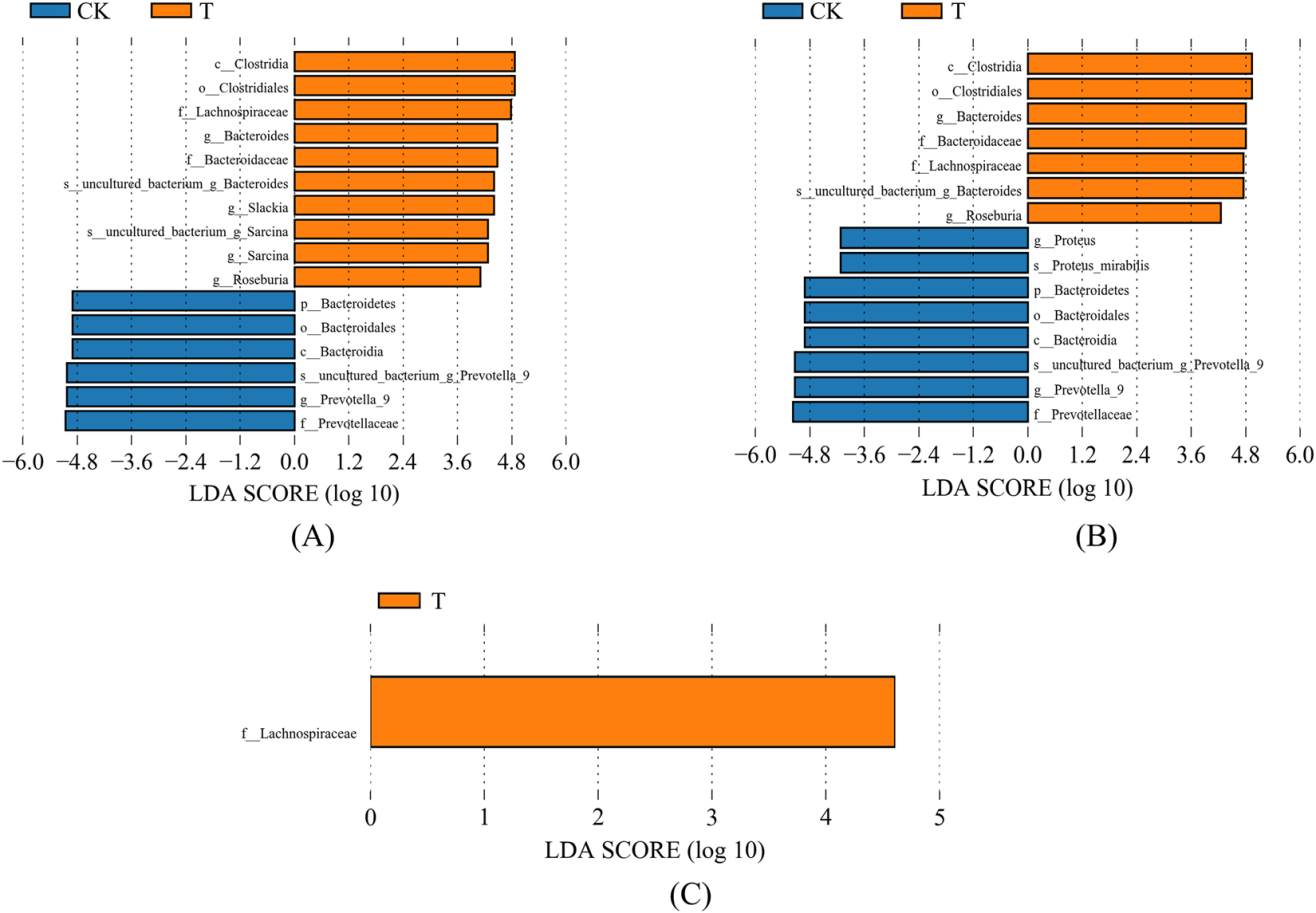
Linear discriminant analysis effect size (LEfSe) cladogram that shows the most differentially abundant taxons between these groups. Taxonomic cladogram obtained from LEfSe analysis of pyrosequencing sequences. Blue indicates taxa enriched in CK, and orange indicates taxa enriched in exposures. Only taxa meeting a linear discriminant analysis (LDA) significant threshold > 4 are shown. (**A**) Taxons significantly different between groups CK and T. (**B**) Taxons significantly different between men from groups CK and T. (**C**) Taxons significantly different between women from groups CK and T.

After species annotation analysis, 490 OTUs were divided into the following seven phyla: *Bacteroidetes*, *Firmicutes*, *Proteobacteria*, *Cyanobacteria*, *Actinobacteria*, *Fusobacteria*, and *Verrucomicrobia* (Fig. 2). *Bacteroidetes* and *Firmicutes* were the most dominant taxa of feces both in T and CK, and their sum was over 90%. This result was consistent with previous reports on the main dominant phyla of *Bacteroidetes* and *Firmicutes* in Chinese people. However, based on the results of the Kruskal-Wallis test, there were no statistical differences in all phyla between the two groups. The same result also occurred for the comparisons between TM and CKM, and between TF and CKF. At the family level, the two groups (T and CK) had twenty equally dominant bacteria (Fig. 3C). The relative abundances of *Lachnospiraceae*, *Erysipelotrichaceae*, *Acidaminococcaceae*, and *Porphyromonadaceae* families in T were significantly higher than those in CK (Fig. 3A). Only the abundance of *Prevotellaceae* showed markedly lower amounts in T (*p* < 0.05). These five families also showed changes in the comparisons between the gender-based groups, although these changes were not statistically significant. In addition, five genera (*Lachnospiraceae*, *Eubacterium eligens*, *Ruminococcaceae* UGG-014, *Erysipelotrichaceae* UCG-003, and *Tyzzerella* 3) presented significantly higher values in T (*p* < 0.05) (Fig. 3B). At the family level, the two groups (TM and CKM, TF and CKF) had twenty equally dominant bacteria (Fig. 4Aa,Ba). However, there were different changes at the genus level revealed by the comparison between groups classified by gender. *Bacteroides*, *Roseburia*, *Pseudobutyrivibrio*, and *Parabacteroides* were enriched in TM, although these changes were not statistically significant (Fig. 4Ab); by contrast, only the relative abundance of *Alloprevotella* was larger in the females in group CK (Fig. 4Bb). Compared with CK, uncultured species in the *Ruminococcus* UCG-014, *Phascolarctobacterium*, and *Tyzzerella* 3 genera exhibited higher values for the T population (Fig. 4Ac,Bc), while only uncultured *Alloprevotella* sp. was found to have a larger abundance in CKF (Fig. 4Bc). A cladogram representative of the structure of the gut microbiota and their predominant bacteria is presented in Fig. 5. The specific bacteria taxa were concentrated in the *Clostridia* and *Bacteroida* classes. Alterations of *Lachnospiraceae*, *Slackia*, and *Prevotellaceae* were consistent with the abovementioned results. However, new findings were also discovered. Greater abundances of *Bacterioides*, *Sarcina*, and *Roseburia* were found in T, which also had a lower abundance of *Prevotella* 9 (Fig. 5A). Similar changes at the class level also occurred, as revealed by the comparison between TM and CKM (Fig. 5B). Intriguingly, *Proteus mirabilis*, the only species in genus *Proteus*, was decreased in TM. In a comparison of the two female groups, only one change happened in the *Lachnospiraceae* family (Fig. 5C). Taken together, the results indicate that presence of multiple metals significantly alters the compositional profile of the gut microbiome.

## Discussion

A healthy microbiota in the intestine can protect against bacterial infection and contribute to intestinal functions including digestion and absorption. Moreover, it is also an effective barrier to protect against the threat of toxic metals^32^. The present study provides novel evidence that long-term exposure to multiple metals including As, Cd, Cu, Pb, and Zn is associated with alterations in the gut microbiome of people living in metal-polluted areas, especially for men.

Metals such as As, Cd, and Cu, as well as excessive amounts of Zn, have been proven to reduce community diversity in previous studies^23,33,34^. Although no effect on α-diversity has been found, Pb was shown to change complexity (β-diversity) in an animal study^35^. However, the results of single exposure in the present study were different from those of previous studies. Multiple metals significantly increased α-diversity in the gut microbiome (Shannon-Wiener and Simpson indices), indicating that the abundance and evenness of bacteria in people who live in the polluted area were higher. Perturbation in the community was also found via the analysis of β-diversity (PCoA and PCA).

Various effects of metals at the phylum level have been found in previous studies; for example, one epidemiological and animal study suggested that early-life lead exposure can significantly affect *Bacteroides* and *Firmicutes* populations, which may be responsible for the expression of the genes involved in lipid metabolites, isooflavone metabolites, and bile acid metabolites^36^. It has also been found by individual studies on As, Cr, and Cd that toxic metal exposure can produce alterations of *Proteobacteria* and *Verrcuomicrobia*^21^. In the results of the present study, the proportions of the three phyla *Bacteroides*, *Firmicutes*, and *Proteobacteria* showed similar changes in people who had suffered from long-term exposure to the metals, albeit without any statistical significance.

There were nine genera that responded obviously to metal exposure, and many of them have proven associations with the functions of the host. Numbers of *Lachnospiraceae* and *Erysipelotrichaceae*, which are linked to intestinal inflammation, were all increased, which is consistent with a previous study on the effect of heavy metals on commensal communities^21^. Another bacterium, *Eubacterium eligens*, which presents a negative correlation with intestinal inflammation, was also enriched in feces from the metal-exposed population. In some previous studies, beneficial bacteria, such as *Roseburia* (associated with effects of colonic motility, immunity maintenance, and anti-inflammatory properties) and *Ruminococcaceae* (mainly associated with mucosa), have presented lower amounts in feces from a population living in a metal-contaminated area^37^. However, in the results of the present study, these populations were all markedly enriched for subjects from the metal-contaminated area. Many studies have reported either reduced abundance of *Prevotella* in relapsing remitting multiple sclerosis (RRMS) patients, or an increased abundance of *Prevotella* after treatment with disease-modifying therapies^38–42^. These data also suggest that *Prevotella* might have an important anti-inflammatory role in RRMS patients. Additionally, Ashutosh *et al*.^43^ reported that *Prevotella* can suppresses disease through the modulation of systemic immune responses. Interestingly, the results of the present study show that an opportunistic pathogen (*Prevotella* 9) was decreased in people burdened with higher metal loads. These people also have a risk of inflammation and other diseases, such as RRMS. This evidence suggests that the homeostasis of gut flora in the residents in the mining and smelting area was broken by the complex toxicity of these metals.

Some differences were also found in the group comparisons based on gender. The same changes of three bacteria (*Bacteroides*, *Roseburia*, and *Prevotella* 9) were found in the male groups. *Proteus*, a gram-negative pathogenic bacterium, was significantly depleted in men who live in the polluted area. All these findings indicate that these metals synergistically operate on the male microbiome. For women, among all the bacteria, only *Lachnospiraceae* showed significant changes. This exceptional gender-based disparity is noteworthy. This is the first time that greater shifts in male microbiome have been demonstrated, and there is unconformity with previous views based on the physical characteristics of women^44^. The lower metal burdens of women living in the contaminated site were proven in the authors’ previous study^31^; this could be the most important reason for the variation of the bacteria in the intestinal tract. The Different living habits (smoking and drinking) and work categories (farming and smelting) between men and females may contribute to this difference. In summation, the intestinal flora in females may retain a large proportion of resistance against long-term metal exposure, while the confusion of the males is already apparent.

The results reveal for the first time that long-term multiple As, Cd, Cu, Pb, and Zn metal exposure can perturb the normal gut community in local inhabitants of contaminated areas, especially in male subjects. Given the importance of gut microbiota to human health, further research is needed to confirm the contribution of the microbiome to metal-related diseases.

## Materials and Methods

### Study area

Two areas from Hezhang County were selected due to their similar geographical factors, life habits, and daily diet. The study area was a Pb-Zn mining and smelting location, called Maomaochang Village (104°10′E to 105°35′E, 26°46′N to 27°28′E), and the surrounding area, Yemachuan Village, was selected as the control area. The distance between Maomaochang Village and Yemachuan is 120 kilometers, and the drinking water of residents come from different sources. Table 3 indicates that the mean values of five metals (As, Cd, Cu, Pb, and Zn) in the plowed soil of the study area were higher than those of the control area, while the Cr and Ni values were similar between the two areas.

**Table 3:** Contents of metals in soils between M and Y (unit: mg/kg).

Element	Mean ± SD	
	_M_a	_Y_b
As	263.17 ± 337.24	22.01 ± 2.82
Cd	46.6 ± 48.1	1.62 ± 0.22
Cr	121.66 ± 36.1	157.5 ± 6.23
Cu	149.84 ± 157.58	79.75 ± 4.58
Ni	50.74 ± 30.92	69.2 ± 3.83
Pb	72.54 ± 33.52	48.71 ± 11.39
Zn	14076.98 ± 11061.7	245.75 ± 11.57

^a^Shao *et al*., 2018; ^b^Briki *et al*., 2016. M: Maomaochang Village; Y: Yemachuan Village.

### Sample collection

In 2016, 85 volunteers took part in a personal information questionnaire. Bowel disease patients, people who had recently used antibiotics or other medicines, and other sick persons were eliminated from among these subjects. Using stool boxes, fecal samples were collected from the remaining subjects in the group. The samples were stored in a 4 °C icebox and then sent back to the lab, after which they were stored at −80 °C in a freezer^45^.

### DNA extraction and 16 S rRNA sequencing

DNA was extracted using the CTAB method with QIAamp genomic DNA and RNA kits (Qiagen, Crawley, West Sussex, UK), and its concentration and purity was monitored on 1% agarose gels. Upon concentration determination, the DNA was diluted to 1 ng/μl and immediately stored at −20 °C. The V3 and V4 regions of 16 S rRNA genes were amplified; the forward primer was CCTAYGGGRBGCASCAG, and the reverse primer was GGACTACNNGGGTATCTAAT. All PCR reactions were carried out with Phusion@ High-Fidelity PCR Master Mix (New England Biolabs). The same volume of 1× loading buffer (containing SYBR green) was mixed with the PCR products, and electrophoresis on 2% agarose gel was carried out for detection. Samples with principal bright bands between 400 and 450 bp were selected for further experiments. Equimolar ratios of the total samples were purified using a Qiagen Gel Extraction Kit (Qiagen, Germany). Sequencing libraries were generated using a TruSeq® DNA PCR-Free Sample Preparation Kit (Illumina, USA) following the manufacturer’s recommendations, and index codes were added; the quality of the library was assessed on Qubit@2.0 Fluorometer (Thermo Scientific) and Agilent Bioanalyzer 2100 systems. Finally, the library was sequenced on an IlluminaHiSeq2500 platform, and 250 bp paired-end reads were generated.

### Bioinformatic analysis

Clean data was attained by jointing raw tags, removing the barcode and primer, and filtering. All effective tags were clustered into operational taxonomical units (OTUs) at the 97% identification level by Uparse v7.0.1001. The Mothur method and SILVA SSU rRNA database were used for species annotation to obtain information on the types of classification levels (Woo *et al*., 2008). Alpha diversity (α-diversity) was obtained to describe the sample complexity; R software (Version 2.15.3) was used to draw the curves of rarefaction and calculate the indices of richness and diversity (ACE, Chao 1, Shannon-Wiener, and Simpson). Excel and SPSS 25.0 were used to evaluate significant differences in these indices. Beta diversity (β-diversity) was used to evaluate the differences between samples in species complexity via unweighted UniFrac distances, which were calculated by QIIME software (Version 1.7.0). Principal coordinate analysis (PCoA) and principal components analysis (PCA) were used to elucidate the distances among the samples. The linear discriminant analysis effect size (LEfSe) and Kruskal-Wallis tests were utilized to analyze the significantly different bacteria.

### Ethical statement

We declare that all experimental protocols were approved by the Human Ethics Committee of China Agricultural University. We also confirm that all methods were performed in accordance with the relevant guidelines and regulations.

### Informed consent

We declare that all the participants were adults. All the subjects knew that drinking water, diet information, urine, and hair samples were collected by our team. We also informed them of the scientific research purposes of all the experimental samples (Appendix S4).

## Supplementary information

Supplementary Figure S1.

Supplementary Figure S2.

The example of Questionnaire.
