# Supplementary material for: Long-term metal exposure changes gut microbiota of residents surrounding a mining and smelting area
Source: Sci Rep. 2020 Mar 10;10:4453. doi: 10.1038/s41598-020-61143-7 (PMC7064573; doi:10.1038/s41598-020-61143-7)
Supplement: Supplementary file 1 — Supplementary Figure S1. [file 41598_2020_61143_MOESM1_ESM.pdf]

Long-term metal exposure changes gut microbiota of residents surrounding a mining and smelting area

Mengmeng Shao,[a,1] Yi Zhu[a]*

[a] The College of Food Science and Nutritional Engineering, China Agricultural University, Beijing 100083, China.

* Corresponding author: Yi Zhu--zhuyi@cau.edu.cn

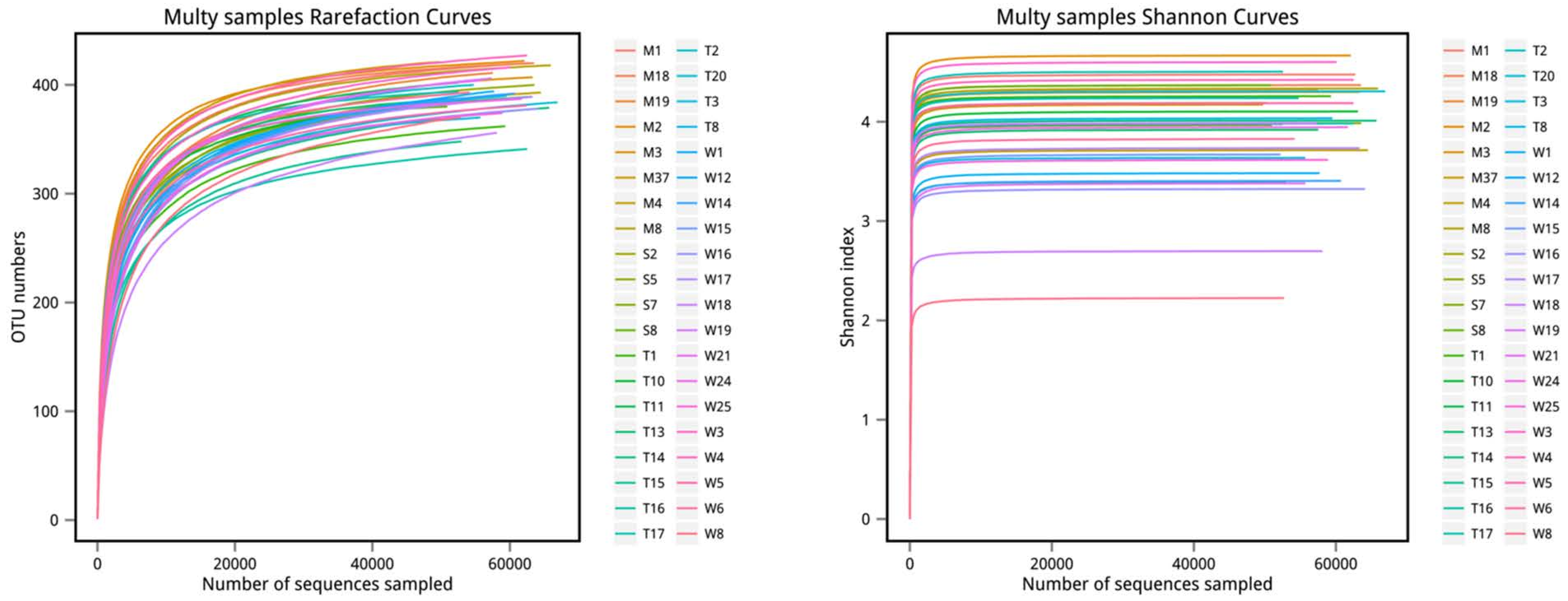
Multy samples Rarefaction Curves
OTU numbers
Number of sequences sampled
0
100
200
300
400
0
20000
40000
60000
M1 M18 M19 M2 M3 M37 M4 M8 S2 S5 S7 S8 T1 T10 T11 T13 T14 T15 T16 T17
T2 T20 T3 T8 W1 W12 W14 W15 W16 W17 W18 W19 W21 W24 W25 W3 W4 W5 W6 W8
Multy samples Shannon Curves
Shannon index
Number of sequences sampled
0
1
2
3
4
0
20000
40000
60000
M1 M18 M19 M2 M3 M37 M4 M8 S2 S5 S7 S8 T1 T10 T11 T13 T14 T15 T16 T17
T2 T20 T3 T8 W1 W12 W14 W15 W16 W17 W18 W19 W21 W24 W25 W3 W4 W5 W6 W8

(A) (B)
