# Supplementary material for: Long-term metal exposure changes gut microbiota of residents surrounding a mining and smelting area
Source: Sci Rep. 2020 Mar 10;10:4453. doi: 10.1038/s41598-020-61143-7 (PMC7064573; doi:10.1038/s41598-020-61143-7)
Supplement: Supplementary file 2 — Supplementary Figure S2. [file 41598_2020_61143_MOESM2_ESM.pdf]

Long-term metal exposure changes gut microbiota of residents surrounding a mining and smelting area

Mengmeng Shao,[a,1] Yi Zhu[a]*

[a] The College of Food Science and Nutritional Engineering, China Agricultural University, Beijing 100083, China.

* Corresponding author: Yi Zhu--zhuyi@cau.edu.cn

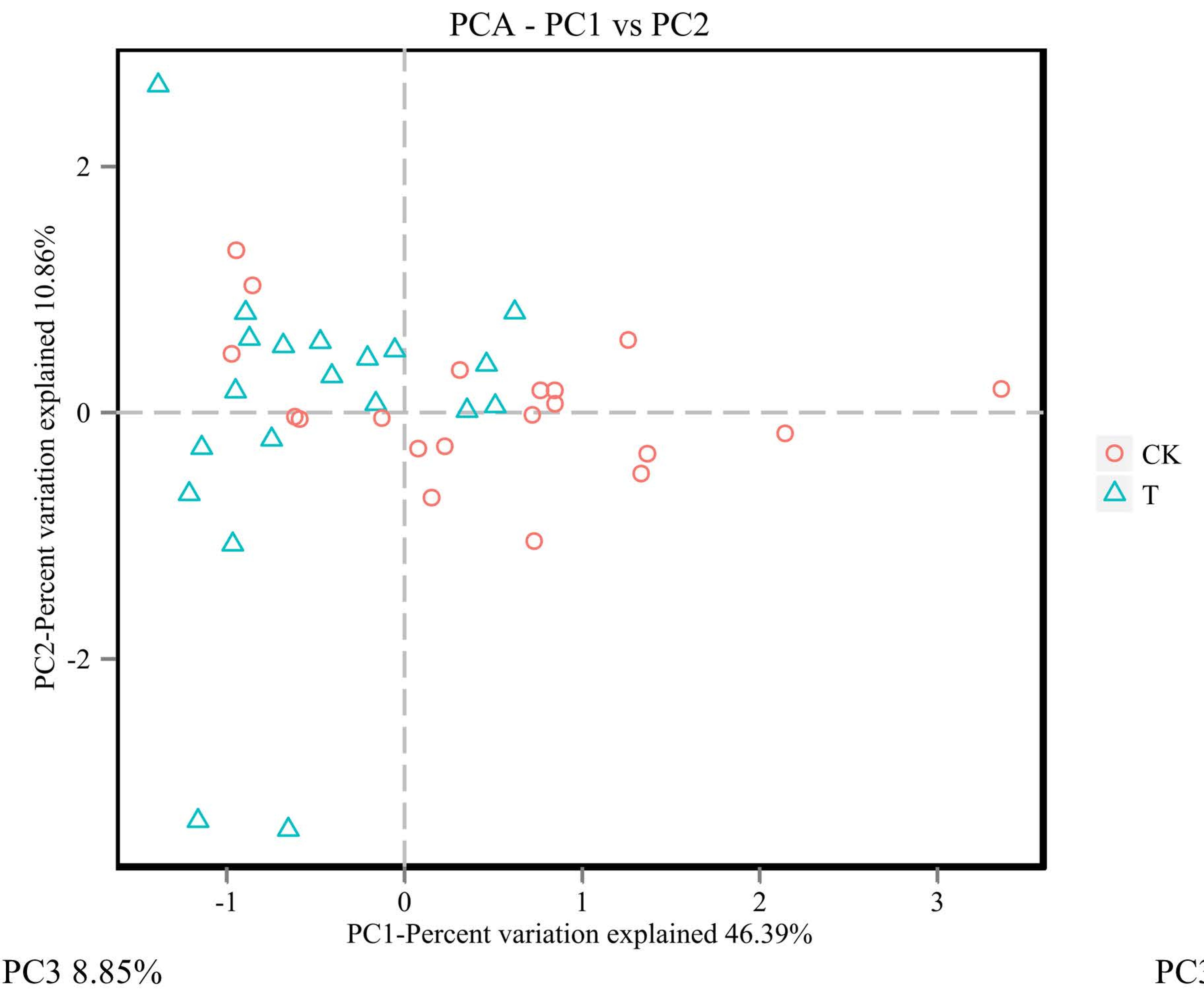

PCA - PC1 vs PC2
PC2-Percent variation explained 10.86%
PC1-Percent variation explained 46.39%
2
0
-2
-1
0
1
2
3
CK
T
PC3 8.85%

(A)

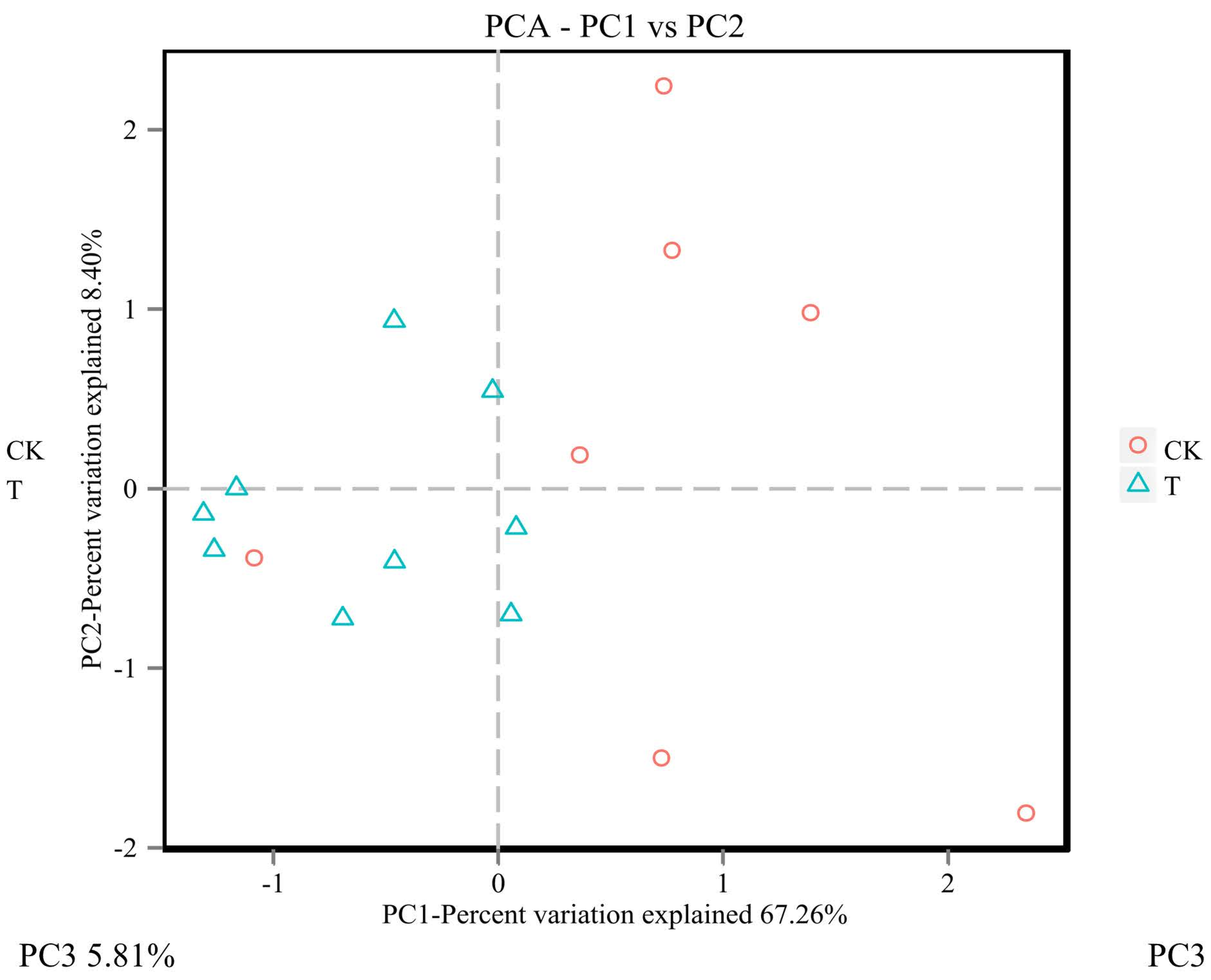

PCA - PC1 vs PC2
PC2-Percent variation explained 8.40%
PC1-Percent variation explained 67.26%
2
1
0
-1
-2
-1
0
1
2
CK
T
PC3 5.81%

(B)

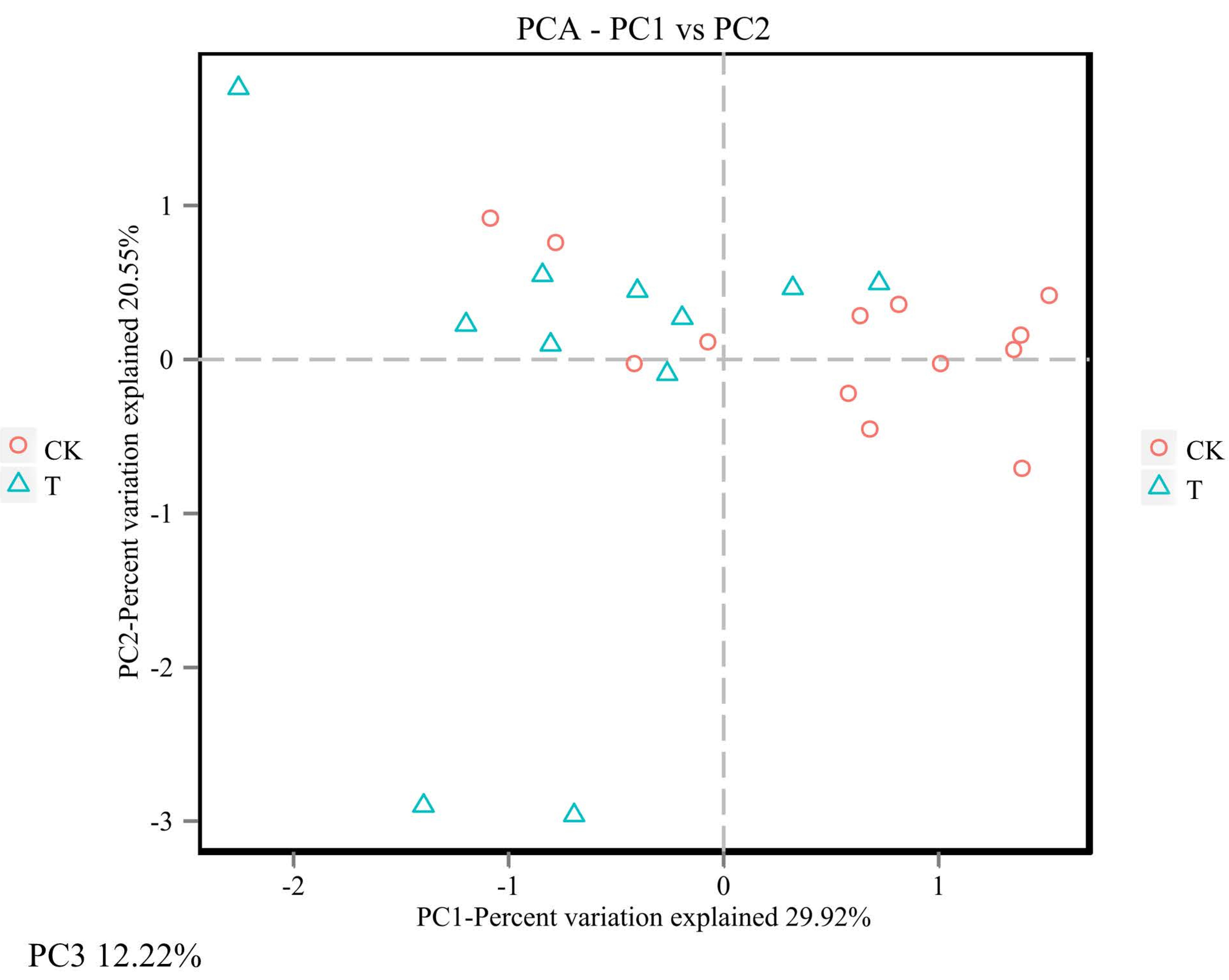

PCA - PC1 vs PC2
PC2-Percent variation explained 20.55%
PC1-Percent variation explained 29.92%
1
0
-1
-2
-3
-2
-1
0
1
CK
T
PC3 12.22%

(C)
