# Supplementary material for: Long-term metal exposure changes gut microbiota of residents surrounding a mining and smelting area
Source: Sci Rep. 2020 Mar 10;10:4453. doi: 10.1038/s41598-020-61143-7 (PMC7064573; doi:10.1038/s41598-020-61143-7)
Supplement: Supplementary file 3 — The example of Questionnaire. [file 41598_2020_61143_MOESM3_ESM.pdf]

Long-term metal exposure changes gut microbiota of residents surrounding a mining and smelting area

Mengmeng Shao,[a,1] Yi Zhu[a]*

[a] The College of Food Science and Nutritional Engineering, China Agricultural University, Beijing 100083, China.

* Corresponding author: Yi Zhu--zhuyi@cau.edu.cn

The example of Questionnaire

1. Basic information: Sex, Age, Weight, Height, Year of Residence
2. Dietary status: Types of food, Intake of various foods
3. Smoking Status
4. Drinking Status
5. Family history
6. Drug use within three months
